# Supplementary material for: Establishment of an Antiplasmodial Vaccine Based on PfRH5-Encoding RNA Replicons Stabilized by Cationic Liposomes
Source: Pharmaceutics. 2023 Apr 12;15(4):1223. doi: 10.3390/pharmaceutics15041223 (PMC10145066; doi:10.3390/pharmaceutics15041223)
Supplement: Supplementary file 1 [file pharmaceutics-15-01223-s001.zip › pharmaceutics-2228425-supplementary.docx]

Supplementary Materials: Establishment of an antiplasmodial vaccine based on PfRH5 encoding RNA replicons stabilized by cationic liposomes

Wesley L. Fotoran, Jamile Ramos da Silva, Christiane Glitz, Luís Carlos de Souza Ferreira and Gerhard Wunderlich

Supplementary Information:


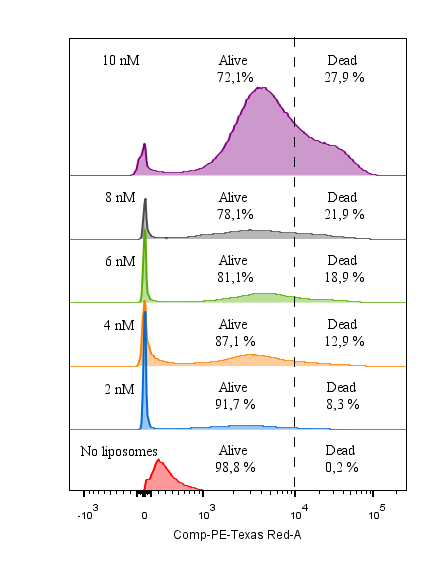


**Figure S1.** Assessment of DDAB-Chol cytotoxicity. Vero cells were incubated with increasing doses of cationic liposomes and incubated for 24 h and then stained with cell imaging stain (LIVE/DEAD™ Cell Imaging Kit Invitrogen) to detect live and dead cells by flow cytometry. The percentages of live and dead cells are shown relative to untreated control (10^4^ fluorescence was considered the threshold for distinguishing live from dead cells). The samples were analyzed in triplicates.

**
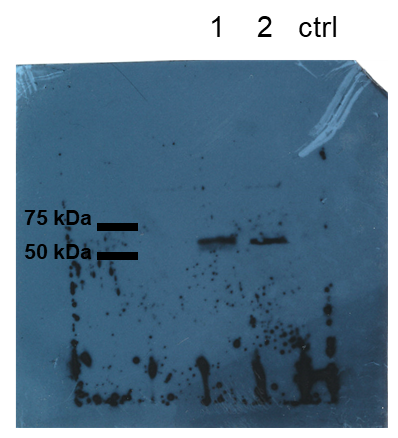
**

**Figure S2.** Original, full size X-ray film of the same Western blot shown in Figure 7. “1“ denotes the detection of an approx. 53 kDa band in NF54 extracts by pooled sera from saRNA-PfRH5 immunized mice, “2“ by pooled serum from TEV-PfRH5 immunized mice, and “ctrl” is the signal obtained by the use of pooled sera from mice before immunization (pre-immune sera). Serum dilution was 1:500 in skimmed milk/Tween 20 (0.05%) and detection was done with a goat anti-mouse HRP antibody (1:2000, KPL).
